# Incomplete cellular reprogramming of colorectal cancer cells elicits an epithelial/mesenchymal hybrid phenotype

**DOI:** 10.1186/s12929-018-0461-1

**Published:** 2018-07-19

**Authors:** Michele Sook Yuin Hiew, Han Ping Cheng, Chiu-Jung Huang, Kowit Yu Chong, Soon Keng Cheong, Kong Bung Choo, Tunku Kamarul

**Affiliations:** 1 Centre for Stem Cell Research & Faculty of Medicine and Health Sciences, Universiti Tunku Abdul Rahman, Sungai Long campus, Bandar Sungai Long, Cheras, 43000 Kajang, Selangor Malaysia; 2 Postgraduate Program, Universiti Tunku Abdul Rahman, Sg. Long, Selangor Malaysia; 3 Department of Animal Science & Graduate Institute of Biotechnology, Chinese Culture University, Taipei, Taiwan; 4 Graduate Institute of Biomedical Sciences, Department of Medical Biotechnology and Laboratory Science & Molecular Medicine Research Center, College of Medicine, Chang Gung University, Tao-Yuan, Taiwan; 5 Dean’s Office, Faculty of Medicine and Health Sciences, Universiti Tunku Abdul Rahman, Sg. Long, Selangor Malaysia; 6 National Orthopaedic Centre of Excellence for Research and Learning & Faculty of Medicine, Universiti Malaya, Kuala Lumpur, Malaysia; 7 Present address: Graduate Institute of Biomedical Sciences, Division of Biotechnology, College of Medicine, Chang Gung University, Tao-Yuan, Taiwan

**Keywords:** Colon cancer, Induced pluripotent cancer cells, Cancer reprogramming, MET and EMT genes, Epithelial-mesenchymal hybrid phenotype

## Abstract

**Background:**

Induced pluripotency in cancer cells by ectopic expression of pluripotency-regulating factors may be used for disease modeling of cancers. MicroRNAs (miRNAs) are negative regulators of gene expression that play important role in reprogramming somatic cells. However, studies on the miRNA expression profile and the expression patterns of the mesenchymal-epithelial transition (MET)/epithelial-mesenchymal transition (EMT) genes in induced pluripotent cancer (iPC) cells are lacking.

**Methods:**

iPC clones were generated from two colorectal cancer (CRC) cell lines by retroviral transduction of the Yamanaka factors. The iPC clones obtained were characterized by morphology, expression of pluripotency markers and the ability to undergo in vitro tri-lineage differentiation. Genome-wide miRNA profiles of the iPC cells were obtained by microarray analysis and bioinformatics interrogation. Gene expression was done by real-time RT-PCR and immuno-staining; MET/EMT protein levels were determined by western blot analysis.

**Results:**

The CRC-iPC cells showed embryonic stem cell-like features and tri-lineage differentiation abilities. The spontaneously-differentiated post-iPC cells obtained were highly similar to the parental CRC cells. However, down-regulated pluripotency gene expression and failure to form teratoma indicated that the CRC-iPC cells had only attained partial pluripotency. The CRC-iPC cells shared similarities in the genome-wide miRNA expression profiles of both cancer and pluripotent embryonic stem cells. One hundred and two differentially-expressed miRNAs were identified in the CRC-iPC cells, which were predicted by bioinformatics analysis be closely involved in regulating cellular pluripotency and the expression of the MET/EMT genes, possibly via the phosphatidylinositol-3 kinases-protein kinase B (PI3K-Akt) and transforming growth factor beta (TGF-β) signaling pathways. Irregular and inconsistent expression patterns of the EMT vimentin and Snai1 and MET E-cadherin and occludin proteins were observed in the four CRC-iPC clones analyzed, which suggested an epithelial/mesenchymal hybrid phenotype in the partially reprogrammed CRC cells. MET/EMT gene expression was also generally reversed on re-differentiation, also suggesting epigenetic regulation.

**Conclusions:**

Our data support the elite model for cancer cell-reprogramming in which only a selected subset of cancer may be fully reprogrammed; partial cancer cell reprogramming may also elicit an epithelial-mesenchymal mixed phenotype, and highlight opportunities and challenges in cancer cell-reprogramming.

**Electronic supplementary material:**

The online version of this article (10.1186/s12929-018-0461-1) contains supplementary material, which is available to authorized users.

## Background

Cellular reprogramming is a de-differentiation process that allows terminally-differentiated cells to acquire pluripotency [1, 2]. To date, numerous somatic cell-derived induced pluripotent stem cell (iPSC) lines have been established via retroviral transfection of the Yamanaka factors, OCT4, SOX2, KLF4 and c-MYC (OSKM) and by other means [3]**.** Induced pluripotent cancer (iPC) cells are iPSCs of cancer origin, which also exhibit the distinctive iPSC characteristics of self-renewal and trilineage differentiation [4]. Diminished cancer phenotypes have also been reported in the iPC cells [5, 6]; reduced in vitro and in vivo tumorigenicity [7] is thought to simulate the early stages of the tumorigenesis [8]**,** opening the possibility that reprogrammed cancer cells may be developed into cancer disease models to recapitulate important early stages in cancer development for mechanistic studies, biomarker discovery and the development of novel therapeutic approaches to treat cancer [8].

In view of accumulated mutations and altered epigenetic programs in cancer cells, significant molecular and biological differences are predicted to exist between somatic and cancer- cell-derived reprogrammed iPSCs and iPCs. MicroRNAs (miRNAs) are important negative regulators of gene expression in most cellular processes [9]**,** and have been implicated in regulating cell fate and maintaining pluripotency [10]. Distinctive miRNA expression profiles have been shown to be associated with OSKM-mediated transcriptional networks and specific signaling pathways in somatic cells [11]. However, systematic studies of miRNA expression and the biological changes elicited by the dysregulated miRNAs in reprogrammed cancer cells are still lacking.

In this study, we first established iPC clones from two colorectal cancer (CRC) cell lines. Using the CRC-iPC cells, we aimed to investigate the pluripotency features of the reprogrammed CRC cells, and possible miRNA involvement in regulating the epithelial-mesenchymal transition (EMT) and the reverse mesenchymal-epithelia transition (MET) processes, both of which are relevant to the establishment and maintenance of pluripotency in reprogrammed normal somatic and cancer cells [12–15]. Surprisingly, our data indicated that the CRC-iPC clones obtained did not attain full pluripotency, and analysis of EMT/MET protein expression further suggested that the partially reprogrammed CRC-iPC cells have acquired an epithelial/mesenchymal (E/M) hybrid phenotype. Our results support the elite model of cancer-cell reprogramming, and highlight intrinsic differences in reprogrammed normal somatic and cancer cells.

## Methods

### Cell lines and culture

A colorectal adenocarcinoma-derived cell line, HCT-15, and a second cell line, SK-CO-1, derived from a metastatic site (ascites) of a 65-year-old patient with colorectal adenocarcinoma, were used in this study. Both cell lines were obtained from ATCC (Manassas, VA, USA). The cell lines were cultured in DMEM high-glucose medium (HCT-15) or MEM (SK-CO-1) supplemented with 10% fetal bovine serum (FBS) (Gibco, Gaithersburg, MD, USA). Human embryonic stem cell (hESC) line H9 and iPC clones were cultured on mitomycin C-treated mouse embryonic fibroblasts (MEF) (Merck Millipore, Darmstadt, German) using a standard hESC medium. For feeder-free culture, cells were passaged to hESC-qualified matrigel (Corning, Avon, NY, USA)-coated culture vessels in mTeSR-1 (Stemcell Technologies, Vancouver, BC, Canada) with daily medium changes. All cultured cell lines were incubated in a humidified atmosphere at 37 ^°^C with 5% CO_2_.

### Retroviral transduction and iPC generation

pMXs retroviral constructs, each harboring one of the *OCT4, SOX2, KLF4* or *c-MYC* genes, were amplified in 293FT cells and the supernatant was filtered through a 0.45-μm pore size PVDF filter as previously described [16, 17]. For retrovirus transduction of the CRC cell lines, the virus supernatant was added to plated CRC cells supplemented with 5 μg/ml polybrene (Merck Millipore, Darmstadt, Germany). The transfected cells were incubated for 24 h before a medium change. Upon reaching confluency, the OSKM-transduced cells were passaged to inactivated MEF. The next day, the medium was replaced with standard hESC medium and cultured using the hESC protocol until the emergence of hESC-like colonies after 21–23 days. Colonies were picked and transferred to fresh MEF feeder layer and continuously cultured in hESC medium [16].

### Immunofluorescence staining

The cells were fixed with 4% paraformaldehyde, incubated at room temperature for 30 min, followed by blocking for 2 h using 1% bovine serum albumin. The cells were washed twice with 1× PBS before addition of primary antibodies of the pluripotency markers, TRA-1-60, TRA-1-81, SSEA-4 or OCT4 (Stemcell Technologies) at 1:100 dilutions and incubated overnight at 4 °C. A FITC-conjugated rabbit anti-mouse antibody (Merck Millipore) was added and the mixture was further incubated for 1 h at room temperature. Nuclei were counterstained with DAPI (Gibco) and observed under an inverted fluorescent microscope.

### In vitro lineage-directed and spontaneous differentiation

Putative CRC-derived induced pluripotent cancer (CRC-iPC) colonies were passaged to a 24-well plate pre-coated with hESC-qualified matrigel, and continuously cultured with osteogenic or adipogenic medium to induce mesoderm differentiation as described [17]. The differentiation medium was changed every alternate day for 21–23 days before staining with Alizarin Red S or Oil Red O (Merck Millipore). For ectoderm-directed differentiation, the putative iPCs were cultured in DMEM/F12 medium, 10% FBS supplemented with 100 ng/ml Noggin (R&D Systems, Minneapolis, MN, USA) for 1 week. For endoderm lineage differentiation, CRC-iPCs were cultured with DMEM/F12 medium with 10% FBS supplemented with 100 ng/ml Activin A (R&D Systems). Ectoderm (MAP2) and endoderm (AFP) (Merck Millipore) markers were also used and observed by immunofluorescence staining. For in vitro spontaneous differentiation, iPCs were cultured in suspension culture in a standard hESC medium for 7 days. The embryoid bodies formed were transferred to 0.1% gelatin coated-culture dish for attachment and further differentiation in FGF-2-free hESC medium. On day 14, the attached embryoid bodies were subcultured to form post-iPC cells in DMEM/F12, 10% FBS and 1% penicillin-streptomycin [5]**.**

### MiRNA microarray and RNA analysis

Total RNA preparations, isolated using the miRNeasy kit (Qiagen, Redwood, CA, USA), were subjected to analysis using Agilent SurePrint Human MiRNA Microarray Release 21.0 (Agilent Technologies, Santa Clara, CA, USA), which consisted of 2549 miRNAs as annotated in miRBase 21. The dataset obtained were analyzed using the GeneSpring GX software version 13.0 (Agilent). Comparative analysis between samples was carried out using the *t*-test (*p*-values) and the Benjamini-Hochberg False Discovery Rate (FDR) correction (adjusted *p*-values) to remove false-positive miRNAs. The criteria set for selection of differentially-expressed miRNAs was log_2_(fold change) ≥ 2 or ≤ − 2 with adjusted *p*-values < 0.05. Hierarchical clustering was performed to identify and visualize pattern of miRNAs expression between samples. The microarray dataset has been deposited with NCBI GEO with the accession number GSE87280. Quantification of mRNA and miRNA expression levels was performed as previously described [18, 19] using primers as shown in Additional file 1: Table S1. For quantitative RT-PCR, the data were analyzed using the comparative ΔΔC_t_ method, normalizing to the GAPDH expression level for mRNAs and that of the RNU6 gene for miRNAs. In all experiments, reactions were performed in triplicates and the data presented as mean ± standard error of the mean derived from three independent experiments. Data were analyzed by Student’s *t* test. *P* < 0.05 was considered statistically significant.

### Western blot analysis

Protein lysates were harvested from the cells using 1× RIPA lysis buffer (Nacalai Tesque, Kyoto, Japan) and kept on ice for 30 min before being centrifuged at 10,000 *g* for 10 min at 4 °C to obtain the supernatant. Protein lysates (50 μg) were subjected to SDS-polyacrylamide gel electrophoresis and western blot analysis as described [19]**.** Dilutions and the sources of the antibodies used are as follows: E-cadherin (CDH1, 1:1000, Cell Signaling Technology), occludin (OCLN, 1:1000, Merck Millipore), vimentin (VIM, 1:1000, Cell Signaling), SNAI1 (1:250, Cell Signaling) and GAPDH (1:2000, Cell Signaling). The protein bands were detected using a horseradish peroxidase-conjugated secondary antibody (1: 10,000, Abcam, Cambridge, UK) for 1 h at room temperature and visualized with the Amersham ECL Western blotting substrate (GE Healthcare), according to the manufacturer’s protocol. The mean of multiple blots is presented (Fig. 4b); the error bars represent standard errors of the mean, SEM.

## Results

### Reprogrammed colorectal cancer (CRC)-induced pluripotent cancer (iPC) cells show ESC-like features and trilineage differentiation

**Fig. 1:**
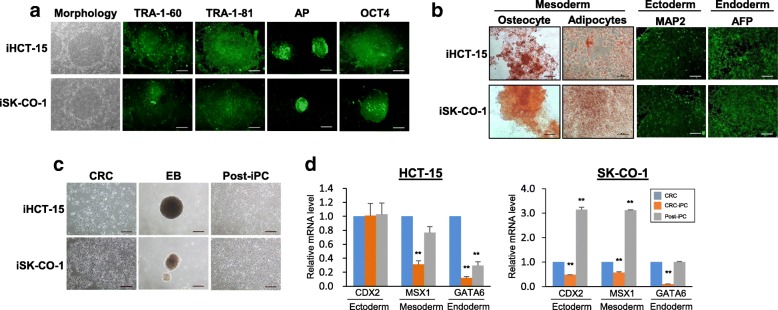
Reprogrammed CRC-iPC colonies show ESC-like features and pluripotency. **a** Morphology and expression of pluripotency markers of the CRC-iPC clones, iHCT-15 and iSK-CO-1. The presence of the pluripotency markers was determined by immunofluorescence staining. **b** Directed-differentiation of iPCs into the trilineage germ layers. For mesoderm lineages, Alizarin Red S and Oil Red O staining were performed; for ectoderm- and endoderm, immunofluorescence detection was used. **c** Spontaneous differentiation of CRC-iPCs. Floating embryoid bodies (EB) were attached and cultured under primary culture conditions to form post-iPCs. Images of the parental CRC morphology are also shown for comparison with that of the post-iPC cells. Scale bars, 100 μm (**a-c**) and 50 μm in oil red O staining of the CRC-iPC cell lines. **d** Expression of germ-layer markers in iPCs and the derived post-iPCs. Data of clone 5 (C5) and 2 (C2) of HCT-15 and SK-CO-1, respectively, are shown. The real-time RT-PCR values were calculated relative to the parental cancer cells and represented as mean ± SEM for triplicate experiments. *p < 0.05 and **p < 0.01

In this study, the colorectal cancer cell lines HCT-15 and SK-CO-1 were used in cellular reprogramming via retroviral transduction of the Yamanaka factors, OCT4, SOX2, KLF4 and c-MYC (OSKM) as previously described [2, 16]**.** The OSKM-transduced cells formed rounded colonies at 21–23 days post-transduction (Fig. 1a). The colonies were picked and were designated as iHCT-15 and iSK-CO-1 for colonies derived from HCT-15 and SK-CO-1, respectively; different independently isolated clones were further designated with the prefix “C” followed by a clone number. Both the iHCT-15 and iSK-CO-1 colonies showed embryonic stem cell (ESC)-like morphology (Fig. 1a, leftmost panels). However, the cells tended to pile up to form multilayers in the center of the putative induced pluripotent cancer (iPC) colonies. Furthermore, the borders of the colonies were not as well-defined as the ESC colonies. The putative CRC-iPCs were stained positive for the pluripotency markers, TRA-1-60, TRA-1-81 and alkaline phosphatase, and a pluripotency marker, OCT4 (Fig. 1a) [20]. When cultured in appropriate differentiation media, the CRC-iPC cells differentiated into osteocyte- and adipocyte-like cells of the mesoderm lineage (Fig. 1b, left panels). In the presence of Noggin and Activin A, the iPCs differentiated towards the ectoderm and endoderm lineages, respectively, as evidenced by immunofluorescence staining of the germ-layer markers, microtubule-associated protein 2 (MAP2, ectoderm) and alpha-fetoprotein (AFP, endoderm) (Fig. 1b, right panels). Using a previously published protocol [5], the CRC-iPCs were also spontaneously differentiated into post-iPCs via embryoid body formation (Fig. 1c, middle and right panels) [5]. The post-iPCs grew in monolayer and were morphologically similar to the parental CRCs (Fig. 1c, left panel). The differentiated post-iPCs re-expressed the germ-layer markers, coudal type homeobox protein 2 (CDX2; ectoderm), Msh homeobox 1 (MSX1; mesoderm) and GATA binding protein-6 (GATA6; endoderm), which were initially significantly down-regulated in the iPC cells on reprogramming (Fig. 1d). The observations suggest reversion to a more primitive state when cancer cells are subjected to reprogramming.

### Down-regulated but reversible expression of pluripotency genes on CRC reprogramming and re-differentiation

**Fig. 2:**
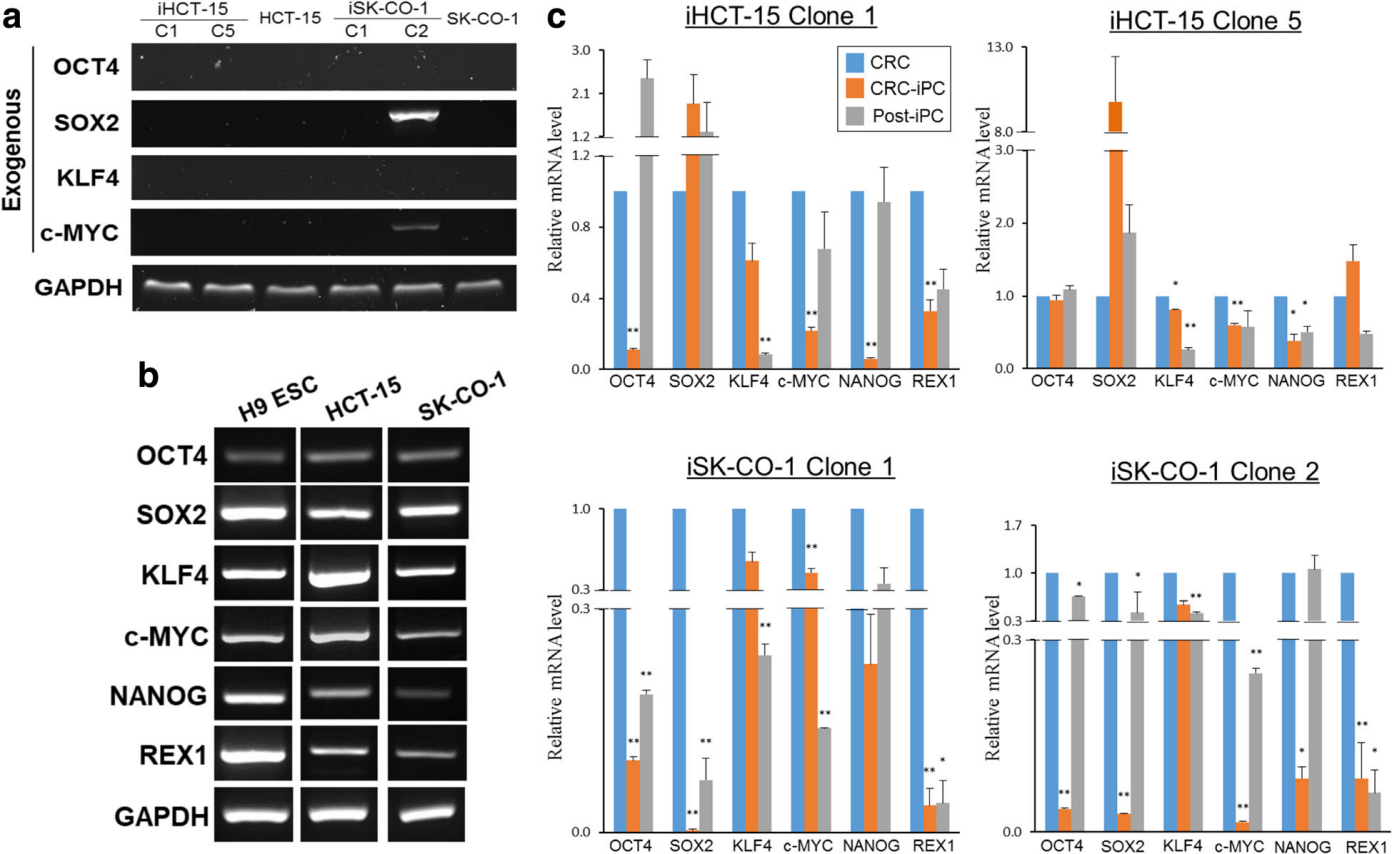
Down-regulated but reversible expression of pluripotency genes on CRC reprogramming and re-differentiation. **a** RT-PCR detection of expression of the exogenous OSKM transgenes. Two independently isolated iPC clones, designated by the prefix “C”, were used. **b** RT-PCR analysis of endogenous expression of selected pluripotency genes in the parental cancer cell lines, and in H9 embryonic stem cells, which was used as a references. In both (**a**) and (**b**), RNA preparations of iPC cells at passage 25 or later were used. **c** Relative expression levels of the pluripotency genes. Expression was quantified by real-time RT-PCR, and the data presented were mean ± SEM from three independent experiments in triplicates. Expression levels of pluripotency genes were normalized to that of the respective parental CRC, which was arbitrarily set as 1.0, *p < 0.05 and **p < 0.01

After *OSKM* transduction, it was important to distinguish between ectopic and endogenous *OSKM* expression. For this purpose, RT-PCR was conducted using primers specific for the transgenes using total RNA isolated from iPC cells at passage 25 or later. In each case, two independent iHCT-15 clones C1 and C5, and iSK-CO-1 clones C1 and C2 were analyzed. The results showed that expression of the transgenes was extinguished with one notable exception **(**Fig. 2a). In iSK-CO-1 clone C2, *SOX2* and *c-MYC* transgene sequences were still detectable, indicating continued ectopic transgene expression. Persistent post-transduction ectopic transgene expression has been reported and interpreted as to indicate incomplete reprogramming [13, 21]. In the RT-PCR analysis, the control H9 embryonic stem cells (ESC) expressed the endogenous levels of the pluripotency genes as anticipated (Fig. 2b). It is, however, noted that the parental HCT-15 and SK-CO-1 cells were already endogenously expressing the pluripotency genes (Fig. 2b), consistent with other reports that pluripotency genes are frequently activated in cancer cell lines [22, 23].

Endogenous expression levels of the pluripotency genes were quantified by real-time RT-PCR (Fig. 2c). Post-iPC cell lines were also included in the pluripotency gene expression analysis. The results showed that, the expression of the pluripotency genes was generally down-regulated in all four CRC-iPC clones, except for *SOX2,* which was up-regulated in the two iHCT-15 clones. Moreover, on re-differentiation to post-iPC cells, expression of the pluripotency genes was generally up-regulated relative to the iPC clones, and often seemed to be reversed back to the parental levels. In the case of *REX1*, re-differentiation did not seem to have altered the expression levels. A notable exception of expression reversal was found in *KLF4* in which its expression was further down-regulated on re-differentiation. Taken together, our data indicated that expression of the pluripotency genes was generally down-regulated on reprogramming, but was not totally shutdown, which raises the possibility of partial or incomplete reprogramming [13, 21]**.** Such down-regulated pluripotency gene expression was reversible, consistent with epigenetic regulation, and was a clear sign of alteration of the cancer phenotype at the molecular level. It is also noteworthy that the CRC-iPC clones thus derived had been maintained for up to 60 passages (data not shown), implying that the maintenance of pluripotency and self-renewal properties of the reprogrammed colorectal cancer cells may not be entirely dependent on the expression levels of the pluripotency genes.

### CRC-iPC cells share similarities in the miRNA expression profiles of both cancer and pluripotent embryonic stem cells

Previous reports have shown that reprogramming alters the miRNA expression profile in induced pluripotent stem cells (iPSCs) derived from both somatic and cancer cells [10, 24]. In addition, miRNA involvement in cellular reprogramming has also been reported [6], indicating important miRNA roles in the induction and maintenance of pluripotency and cell fate [19, 25, 26]. However, there are few studies on genome-wide miRNA expression profiling in reprogrammed cancer cells. For this purpose, total RNA preparations of the HCT-15 and SK-CO-1 cells and two iPC clones from each cell line were subjected to miRNA microarray analysis (NCBI GEO accession number: GSE87280). A human ESC cell line, H9, was also included as a reference cell line.

**Fig. 3:**
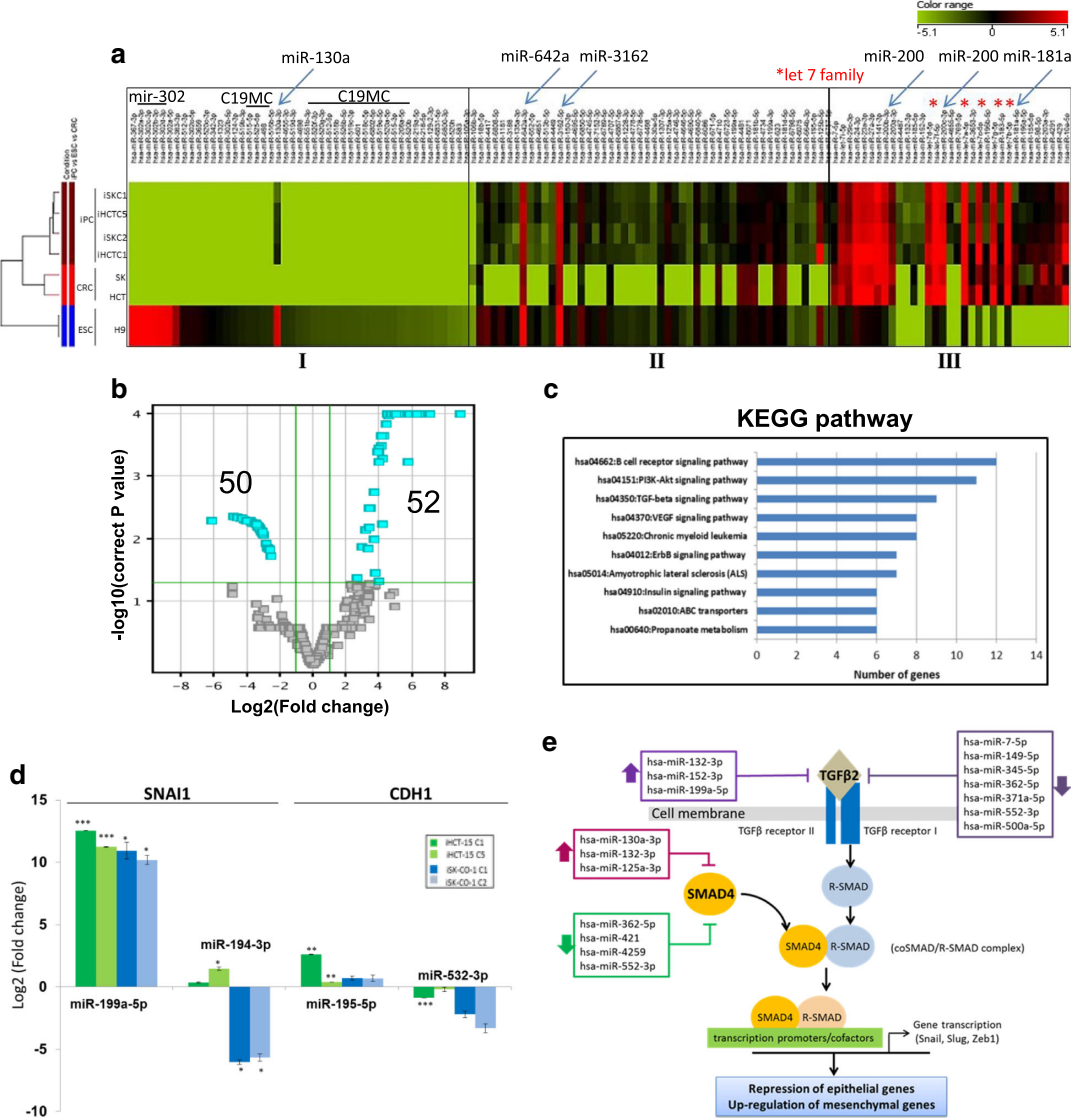
Reprogramming-induced differential expressed miRNAs in CRC-iPC cells regulate EMT via the TGF-β signaling pathway. **a** Hierarchical clustering analysis of CRC-iPCs, the parental CRCs and an ESC line, H9, in duplicate. The differentially-expressed miRNAs are categorized into three groups, I – III. MiRNAs that have been previously reported to be associated with reprogramming are shown on the top: horizontal bars indicate miRNA families; blue slanting arrows indicate the individual miRNAs and red asterisks indicate the let 7 miRNA family (see Additional file 2: Table S2 and text for further description). Color scale bar indicates the relative miRNA expression level. **b** Volcano plot identified 50 and 52 down- and up-regulated miRNAs (light blue-colored cubes) in the CRC-iPC cells with log_2_(fold change) ≥ 2.0 or ≤ − 2, and *p*-value < 0.05. **c** KEGG pathway analysis of the predicted target genes. **d** Expression validation of randomly-selected miRNAs targeting *SNAI1* and E-cadherin (*CDH1*) (see Table 2) by real-time RT-PCR. The data shown are the mean values obtained from the four iPC clones analyzed in the study. Expression levels were relative to the parental cells, represented as mean ± SEM; *p < 0.05, **p < 0.01, ***p < 0.001. **e** Predicted targeting of the TGF-β pathway that regulates the EMT/MET processes by the differentially expressed miRNAs. The predicted miRNAs are shown in boxes; up- and downward thick arrows indicate the up- or down-regulated miRNAs. The TGF-β pathway was modified from [38]

Hierarchical clustering analysis of the miRNA profiles showed that the differentially-expressed miRNAs could be grouped into three major clusters based on their expression patterns (Fig. 3a; see also Additional file 2: Table S2). Cluster I miRNAs were highly or moderately expressed in ESC cells, consistent with the inclusion in this cluster the known reprogramming miR-130 [27], the mir-302 family (miR-302a, −302b, −302c & -302d [28, 29], and the chromosome 19 miRNA cluster (C19MC) miRNAs, which have previously been shown to modulate the early phases of reprogramming ([19]) (Fig. 3a & Additional file 2: Table S2). On the other hand, the cluster I miRNAs were not expressed, or expressed at low levels, in the parental CRC cells, and that these miRNAs remained unexpressed on reprogramming. Cluster II consists of miRNAs that were unexpressed, or expressed at low expression levels, in CRC cells, but were up-regulated in the iPC cells to levels comparable to H9 ESC. However, some cluster II miRNAs, including miR-642a and − 3162-5p, were already highly expressed in CRC, and remained consistently expressed iPC cells as were in the ESC cells (Fig. 3a & Additional file 2: Table S2). The results suggest that cluster II miRNAs are associated with pluripotency, but are already activated in CRC cells. Cluster III includes miRNAs that were already highly expressed in the parental CRC cells and expression levels remained high, or were up-regulated on reprograming in the CRC-iPC cells to levels higher than in the ESC cells, which showed only low expression levels, suggesting that these miRNAs are associated more with tumorigenesis than with the reprogramming process. Cluster III includes the let-7 and miR-200 (miR-200a,-200b,-200c) families (Fig. 3a & Additional file 2: Table S2). The let-7 family suppresses pluripotency by modulating expression of pluripotency genes, including *c-MYC* and *Lin28* [30], whereas the miR-200 cluster is important in the early stage of somatic cell reprogramming by activating mesenchymal-to-epithelial transition (MET) [31].

Taken together, miRNA expression profiling revealed that the reprogrammed CRC-iPC cells retained some molecular signatures of the parental cells, indicating retention of cancer phenotype at the molecular level. On the other hand, despite the demonstrated pluripotency, CRC-iPC cells showed distinct dissimilarity with the pluripotent ESC cells, hinting incomplete reprogramming due to the tumor phenotype of CRC.

### Reprogramming activates miRNAs targeting the TGF-β cell signaling pathway to regulate expression of EMT/MET genes

MiRNA profiling showed that the miRNA expression profiles were highly similar between the four iPC clones, irrespective of the parental sources (Fig. 3a). Using a stringent selection criteria of log_2_(fold change) (FC) ≥ 2.0 or ≤ − 2.0 and *p*-value < 0.05, a total of 102 statistically significant differentially-expressed miRNAs were thus identified, fifty of which were down-regulated and fifty-two miRNAs were up-regulated (Fig. 3b; for a full miRNA list, see Additional file 3: Table S3). The top ten most significantly up- or down-regulated miRNAs are shown in Table 1. Interestingly, three of the top ten up-regulated miRNA in the CRC-iPC cells are mapped in the highly unstable regions of the 19p13.13 and 19q13.11 deletion syndromes [32, 33], whereas the gene encoding miR-362-5p in the down-regulated group is mapped at Xp11.23 associated with the Xp11.22-Xp11.23 duplication syndrome [34]. About half of the top 10 miRNAs have not been annotated.

**Table 1:** Top ten differentially up- or down-regulated miRNAs in reprogrammed CRC-iPCs

miRNA	Chromosome	miRNA family^a^	Log_2_FC^b^
A. Up-regulated			
miR-125b-5p	11q24.1	mir-10	8.88
miR-199a-3p	19p13.2	mir-199	7.04
miR-125a-3p	19q13.41	mir-10	6.89
miR-4734	17	NA	6.36
miR-6789-5p	19	NA	6.24
miR-4417	1	NA	6.21
miR-150-3p	19q13.33	mir-150	5.86
miR-6723-5p	1	NA	5.77
miR-3934-5p	6	mir-3934	5.71
miR-1181	19	mir-1181	5.67
B. Down-regulated			
miR-192-5p	11q13.1	mir-192	−6.17
miR-338-3p	17q25.3	mir-338	−4.84
miR-455-3p	9q32	mir-455	−4.63
miR-362-5p	Xp11.23	mir-362	−4.42
miR-6741-3p	1	NA	−4.37
miR-6743-3p	11	NA	−4.34
miR-552-3p	1p34.3	mir-552	−4.15
miR-6782-5p	17	NA	−4.05
miR-4254	1	NA	−4.02
miR-4725-5p	17	NA	−3.99

^a^Based on miRBase database ver. 21; Log_2_ Fold change (FC) is relative to the parental CRC cells. Only log_2_FC > 2.0 or < − 2.0 and *p* < 0.05 are shown. *NA* not annotated

To elucidate possible functional roles, putative targets of the differentially expressed miRNAs were identified by interrogating the TargetScan and MicroRNA.org databases and the Kyoto Encyclopedia of Genes and Genomes (KEGG) pathways were mapped, which showed that six of the top 10 KEGG pathways were related to cellular signaling (Fig. 3c), including the TGF-β pathway involved in the regulation of the EMT processes [35]. MET is a critical step in setting up the initial phase of somatic cell reprogramming; hence, the expression status of MET and EMT genes is an important indicator of the cell state [36]. On database interrogations, 25 of the differentially expressed miRNAs, both in the up- and down-regulated groups, were found to target transcripts of two representative EMT genes, vimentin (*VIM)* and Snail1 (*SNAI1)* and two typical MET genes, E-cadherin (*CDH1)* and occludin (*OCLN*) (Table 2). For validation, miRNAs targeting *SNAI1* and *CDH1* were randomly chosen for qRT-PCR analysis (Fig. 3d). The predicted expression was largely consistent with the exception that miR-194-3p was slightly up-regulated in the HCT-15 iPC clones when down-regulation was shown in microarray analysis. The minor discrepancy may be explained by the fact that in the miRNA profiling analysis (Table 1 and Additional file 2: Table S2), mean values of four iPC clones from the two parental CRC cell lines were derived, whereas individual iPC clones were subjected to qRT-PCR analysis (Fig. 3d).

**Table 2:** Differentially expressed miRNAs in the iPC clones targeting selected mesenchymal and epithelial protein genes

Gene	miRNA^a^	Chromosome	miRNA family	Log_2_(FC)^b^
EMT genes				
*VIM*	miR-4745-5p	19	NA	5.61
	miR-3188	19	mir-3188	4.55
	miR-30a-5p	6q13	mir-30	3.94
	miR-500a-5p	Xp11.23	mir-500	−2.85
	miR-7-5p	15q26.1	mir-7	−2.61
	miR-335-3p	7q32.2	mir-335	−2.61
*SNAI1*	miR-199a-5p	19p13.2	mir-199	5.06
	miR-4695-5p	1	NA	4.44
	miR-30a-5p	6q13	mir-30	3.94
	miR-194-3p	11q13.1	mir-194	−3.03
MET genes				
*CDH1*	miR-671-5p	7q36.1	mir-671	5.20
	miR-769-5p	19q13.32	mir-769	5.02
	miR-4695-5p	1	NA	4.44
	miR-195-5p	17p13.1	mir-15	3.96
	miR-4725-5p	17	NA	−3.99
	miR-532-3p	Xp11.23	mir-188	−3.64
	miR-362-3p	Xp11.23	mir-362	−3.60
	miR-149-5p	2q37.3	mir-149	−3.36
	miR-335-3p	7q32.2	mir-335	−2.61
*OCLN*	miR-1228-3p	12	mir-1228	4.75
	miR-132-3p	17p13.3	mir-132	4.20
	miR-4463	6	NA	3.40
	miR-513b-5p	Xq27.3	mir-506	3.38
	miR-362-5p	Xp11.23	mir-362	−4.42
	miR-3591-3p	18	mir-122	−3.97
	miR-362-3p	Xp11.23	mir-362	−3.60
	miR-449b-3p	5q11.2	mir-449	−3.39
	miR-500a-5p	Xp11.23	mir-500	−2.80
	miR-335-3p	7q32.2	mir-335	−2.61

^a^Taken from Additional file 2: Table S2. Target genes were predicted by one or more of the databases TargetScan 7.0, miRWalk2.0 and Diana tools (microT-CDS). ^b^Log_2_(fold change) data are presented in decreasing values; miRNAs that were down-regulated are shown as negative values. All values are statistically significant with *p* < 0.01. *NA* not available

Target prediction data (Table 2) showed that each of the four EMT/MET genes was targeted by multiple miRNAs that were both up- or down-regulated on reprogramming. Some miRNAs also targeted two or more genes. Notably, miR-335-3p was down-regulated on reprogramming and the miRNA was predicted to target an EMT gene *SNAI1*, and both the MET *CDH1* and *OCLN* genes (Table 2). Both the miR-362-5p and -3p species, which have different sequences, were down-regulated and targeted different EMT and MET gene.

The TGF-β pathway, which has previously been shown to regulate the EMT/MET processes [35], is one of the top ten predicted KEGG pathways targeted by the reprogramming-induced dysregulated miRNAs (Fig. 3c) To further establish possible relationship between the miRNAs and the TGF-β pathway, targeting miRNAs were mapped to the major components of the pathways in relation to EMT regulation (Fig. 3e). In this study, bioinformatics analysis predicted that TGFβ2, a ligand of the TGFβ family [37], was regulated by ten miRNAs, seven of which were down-regulated and three were up-regulated on reprogramming (Fig. 3e). Furthermore, SMAD4, which complexes with phosphorylated R-SMAD further to activate or enhance transcription of downstream effector genes [38], was also predicted to be targeted by seven differentially expressed miRNAs. The down-regulation of these miRNAs may up-regulate TGFβ2 and SMAD4 expression, the TGF-β signaling cascade and expression of the EMT-associated genes, *SNAI1*, *SLUG* and *ZEB1*, resulting in a cell transition towards the mesenchymal-like state [38]. In addition, up-regulated *SNAI1* expression may also lead to repression of the epithelial E-cadherin and occludin proteins, resulting in the reversal of the MET process [39]. Taken together, many of the cancer cell-reprogramming-induced dysregulated miRNAs are predicted to be associated with EMT/MET regulation, and subtle interplay between the up- and down-regulated miRNAs may decide the predominance of either or both the EMT and MET processes.

### Dysregulated expression of EMT and MET proteins on reprogramming suggests an E/M hybrid phenotype

**Fig. 4:**
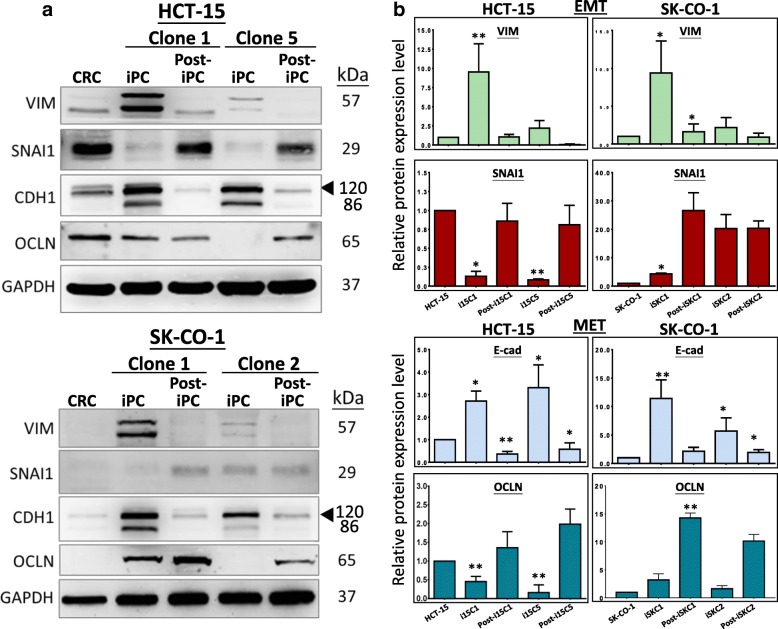
Reversible dysregulated expression of MET and EMT proteins on CRC reprogramming and re-differentiation. **a** Representative western blots of the EMT proteins, vimentin (VIM) & Snail1 (SNAI1) and the MET proteins E-cadherin (CDH1) and occludin (OCLN) in the parental CRC, the iPC and the respective differentiation-derived post-iPC clones. In the E-cadherin blots, arrowheads indicate the anticipated protein size (see text). **b** Quantification of changes of EMT and MET protein levels from three independent experiments. Data presented as mean ± SEM. *P* values were derived by comparing iPC vs parental cells and post-iPC vs iPC cells; *p < 0.05; **p < 0.01

Effects of reprogramming on EMT/MET gene expression in CRC cells were next investigated by western blot analysis in the iPC and post-iPC cells, focusing on the same EMT proteins, vimentin and Snail1, and MET proteins, E-cadherin and occludin, examined above. Representative western blots are shown in Fig. 4a; quantification of the data from three independently performed western blots is shown in Fig. 4b. Vimentin levels were low in the parental HCT-15 and SK-CO-1 cells. On reprogramming, the protein levels were up-regulated between ~ 2.5- to ~ 8-fold in the different iPC clones of both cell lines. On differentiation into post-iPC clones, vimentin levels were generally suppressed or reverted back to levels similar to those of the parental cells. SNAI1 level was high in HCT-15 but was very low in SK-CO-1. On reprogramming, the SNAI1 levels were suppressed in the iHCT-15 clones, but were up-regulated to various extents in the two iSK-CO-1 clones. On differentiation, the SNAI1 levels were restored to approximately the parental levels in post-iHCT-15 clones, but the protein was further up-regulated in post-iSK-CO-1 cells. In the E-cadherin western blots, a 120-kD band that was detected all cell types (Fig. 4a, E-cadherin, arrowhead); however, a second 86-kD species appeared in the iPC cells, which was most likely the E-cadherin soluble ectodomain, a cleavage product of matrix metalloproteinases of the 120-kD protein [40, 41]. E-cadherin was consistently up-regulated in all iPC clones from the two different cell lines; on differentiation, the protein levels were all reverted back to a level comparable to that of the parental CRC cells. Expression of occludin was clearly detected in the HCT-15 cells, but the protein was present only in a very low level in SK-CO-1. Occludin levels were down-regulated in the iHCT-15 iPC clones, and were restored to approximately the parental levels on differentiation. On the other hand, occludin was up-regulated in the reprogrammed iSK-CO-1 cells, and differentiation further up-regulated the protein to higher levels.

Paradoxically, the western blot data revealed that on reprogramming, both the EMT and MET proteins were generally up-regulated, with exception in the SNAI1 and, to a lesser extent, occludin, in the iHCT-15 cells. Hence, the conflicting EMT and MET expression patterns suggested the possibility that reprogramming of cancer cells might have elicited an epithelial/mesenchymal (E/M) hybrid phenotype [42, 43]. The data also showed that initial differentiation of the iPC clones generally reversed expression of the EMT/MET protein, echoing epigenetic regulation, but with the exception of SNAI1 and occludin in the reprogrammed iSK-CO-1 clones. Different clones of the same cell line, and different CRC cell lines also showed different EMT/MET expression profiles, which could be explained by cellular heterogeneity and cancer stage-dependent expression modes of the EMT/MET genes in the cancer cells [44, 45].

## Discussion

In this work, the OSKM-reprogrammed CRC-iPC cells were able to undergo in vitro tri-lineage differentiation showed other pluripotency characteristics similar to those of embryonic stem cells. However, several lines of experimental evidence suggested that the CRC-iPC clones obtained had not undergone complete reprogramming. Firstly, the border of the iPC colonies formed was not well-defined, and the cells piled up in the center (Fig. 1a). Secondly, in some CRC-iPC clones, ectopic expression of the transgenes had not been completely extinguished (Fig. 2a). In other reports, partially-reprogrammed somatic cells, which are also called pre-iPSC, are often characterized by low levels of endogenous pluripotency genes while the exogenous transgenes may still be sustained in the pre-iPSC [13, 21], as observed in this work for the iSK-CO-1 clone 2, which failed to attenuate ectopic expression of *SOX2* and *c-MYC* (Fig. 2a), suggesting incomplete reprogramming. However, residual transgene expression is not specific to retroviral transduction method [46] since OSKM-retroviral transduced sarcoma [7] and colorectal cancer cells [5] frequently showed efficient and complete silencing of the OSKM transgenes. A third line of evidence to support incomplete reprogramming is that the reprogrammed CRC-iPC cells showed distinct dissimilarity in the miRNA profiles compared with the pluripotent H9 ESC cells, while retaining similarity in the molecular signature with the parental cancer cells (Fig. 3a). Furthermore, the CRC-iPC cells failed to form teratoma in vivo in nude mice (Choo et al., unpublished data;). Indeed, others have also shown that reprogrammed cancer cells frequently fail to form teratoma, suggesting that limited pluripotency may be a common feature in reprogrammed cancer cells [26, 47]. In addition, members of the miR-302/− 367 families, which were previously used to generate iPSCs and iPCs [48], were not found among the 102 differentially-expressed miRNAs in the CRC-iPCs generated (Additional file 3: Table S3). On the contrary, the miRNA expression profiles indicated miRNA involvement in the regulation of EMT (Table 2 and Fig. 3e), which raises the possibility of an alternate route in cancer cell-reprogramming as discussed above**.**

Furthermore, the six pluripotency genes examined, including *OSKM, NANOG* and *REX1*, are already expressed in the parental CRC cells, as reported for other cancer cells [22, 23]. In the reprogrammed CRC-iPC cells, expression of these genes was generally down-regulated, which was reversible on re-differentiation (Fig. 2). Others have shown down-regulation, or limited activation, of endogenous pluripotency genes, in somatic cell reprogramming [14].

**Fig. 5:**
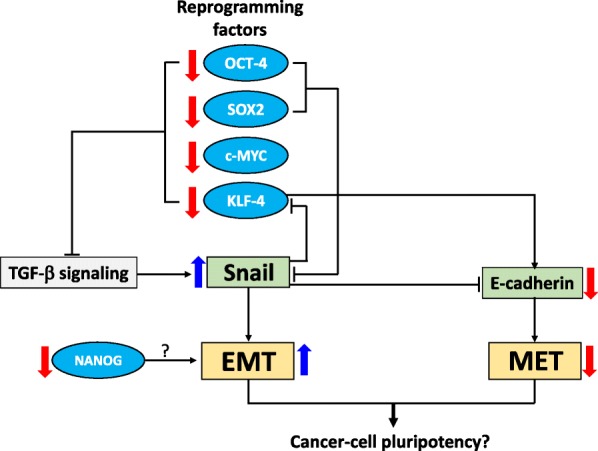
A proposed scheme that links down-regulated expression of pluripotency genes with cancer cell reprogramming. See Discussion for description of the scheme

The finding that endogenous expression of the pluripotency genes was down-regulated on CRC reprogramming may, in fact, be linked to the epithelial or mesenchymal state of the reprogrammed cells, as proposed in the scheme shown in Fig. 5. OCT4 and SOX2 have previously been shown to inhibit expression of the EMT-inducing factor, SNAI1, which works synergistically with KLF4 to induce an epithelial phenotype by up-regulating E-cadherin [49]. However, KLF4 is, in turn, negatively regulated by SNAI1 [50]. Thus, the up-regulation of SNAI1 may have inhibited KLF4, leading to down-regulation of E-cadherin and, hence, the suppression of MET. However, it is unclear how down-regulation of NANOG could promote EMT. In addition, the core pluripotency genes *OSKM* have previously been shown to inhibit various factors of the TGF-β signaling pathway, and that OCT4 and SOX2 suppress SNAI1 [49]. Hence, *OSKM* down-regulation is likely to have contributed to the TGF-β signaling pathway to up-regulate SNAI1 expression and, therefore, promoting EMT (Fig. 5). Pluripotency gene-modulated transition between MET and EMT may, therefore, play a vital role in inducing pluripotency in cells of cancer origin.

The strongest evidence to suggest partial reprogramming came from the expression analysis of the EMT/MET genes in the reprogrammed iPC cells (Fig. 4). As pointed out above, previous studies have shown that MET is an essential early step in somatic cell reprogramming [14, 51], whereas EMT counterbalances the MET effects to hinder reprogramming [52]. In the reprogrammed CRC-iPC clones, the four EMT and MET proteins investigated were not consistently negatively or positively regulated on reprogramming to clearly confer either an epithelial or mesenchymal phenotype to the iPC cells, and the reprogramming-induced alterations in EMT and MET gene expression were generally reversible (Fig. 4). The western blot data could be interpreted to indicate that the reprogrammed cancer cells had acquired an intermediate state of an epithelial/mesenchymal (E/M) hybrid phenotype [53], as has previously been reported in adult epithelial, hepatic and neoplastic stem cells [42]. Others have also developed models to suggest that such a mixed E/M phenotype is associated with multiple stemness characteristics of cancer stem cells, including tumor initiating and self-renewal abilities [42, 54, 55].

Increasing evidences are also suggesting that EMT may also be an important participant in regulating cellular reprogramming [56]. Sequential introduction of the OSKM factors induced an early and transient response of EMT prior to the appearance of a delayed MET response in reprogrammed somatic cells [12]. Hence, beside the proposed E/M hybrid phenotype, there may also exist a delicate and dynamic balance between EMT and MET per se to confer either an EMT or MET phenotype to reprogrammed cancer cells. In terms of miRNA regulation, the four MET/EMT genes analyzed here are also targeted by miRNAs both up- or down-regulated on reprogramming (Table 2 & Fig. 4). Hence, expression of the EMT/MET proteins in the iPC cells may further dependent on delicate interplays between multiple targeting miRNAs and other regulatory factors.

Zhang et al. (2013) have previously proposed a pluripotency hierarchy model in which the cancer cell-derived iPCs are thought to be induced to a pre-iPSC state, just a level above the multipotent mesenchymal stem cells [7]. The pre-iPSC state of reprogrammed cancer cells is also thought to be reflected in the down-regulation of pluripotency genes [7]. Hence, reprogramming of normal somatic cells follows a continuous stochastic model, where all transduced cells have equal probability to be transformed into a pluripotent state [57, 58]. In contrast, evidences have been presented to support the elite model for cancer-cell reprogramming in which only a selected subset of cells in the heterogeneous cancer cell population may be fully reprogrammed into iPCs [59]. Our work here highlights challenges and in fully reprogramming cancer cells.

## Conclusions

In this work, Yamanaka factor-reprogrammed colorectal cancer-induced pluripotent cancer cells show ESC-like features and trilineage differentiation. Down-regulated expression of pluripotency genes and dissimilar miRNA expression profiles to that of pluripotent embryonic stem cells indicate incomplete reprogramming. Reprogramming activates miRNAs that target the TGF-β signaling pathway to modulate expression of EMT/MET genes; however, dysregulated expression of EMT and MET proteins suggests an epithelial-mesenchymal hybrid phenotype, consistent with partial CRC reprogramming. Our data further highlight challenges in obtaining fully reprogrammed cancer cells likely due to the accumulated mutations and epigenetic modifications in the cancer cells. Nonetheless, the reprogrammed CRC-iPC cells offer an opportunity for the development of disease models to further elucidate the molecular mechanism in the tumorigenesis of colorectal cancer.

## Additional files

Additional file 1: **Table S1.** Primer sequences of genes and miRNAs analyzed in RT-PCR or qRT-PCR. (PDF 99 kb)

Additional file 2: **Table S2.** Regulated expression of miRNAs known to modulate cellular reprogramming. (PDF 38 kb)

Additional file 3: **Table S3.** Full list of 102 differentially expressed miRNAs in 4 iPCs vs 2 CRCs. (PDF 220 kb)

## Abbreviations

CDH: E-cadherin
CDX2: Coudal type homeobox protein 2
CRC: Colorectal cancer
EB: Embryoid body
EMT: Epithelial-mesenchymal transition
ESC: Embryonic stem cells
GAPDH: Glyceraldehyde-3-phosphate dehydrogenase
GATA6: GATA binding protein-6
iPC: Induced pluripotent cancer
iPSC: Induced pluripotent stem cell
MAP2: Microtubule-associated protein 2
MEF: Mouse embryonic fibroblasts
MET: Mesenchymal-epithelial transition
MSX1: Msh homeobox 1
OCLN: Occludin
OSKM: OCT4, SOX2, KLF4 and c-MYC
SNAI1: Snail1
VIM: Vimentin
