# Supplementary material for: Incomplete cellular reprogramming of colorectal cancer cells elicits an epithelial/mesenchymal hybrid phenotype
Source: J Biomed Sci. 2018 Jul 19;25:57. doi: 10.1186/s12929-018-0461-1 (PMC6052640; doi:10.1186/s12929-018-0461-1)
Supplement: Supplementary file 1 — Table S1. Primer sequences of genes and miRNAs analyzed in RT-PCR or qRT-PCR. (PDF 99 kb) [file 12929_2018_461_MOESM1_ESM.pdf]

Additional file 1: Table S1. Primer sequences of germ-layer markers and pluripotency genes analyzed in RT-PCR or qRT-PCR

Genes	Primer Sequences (5'-3')	Base pair size (bp)
	**Germ-layer markers**	
CDX2	F: AAAGGCTTGGCTGGTGTATG<br>R: GTCAGGCCTGGAGTCCAATA	66
GATA6	F: TCTACAGCAAGATGAACGGCCTCA<br>R: TCTGCGCCATAAGGTGGTAGTTGT	125
MSX1	F: GAGTTCTCCAGCTCGCTCAG<br>R: TCTCCAGCTCTGCCTCTTGT	100
	**Pluripotency transgenes (**Takahashi and Yamanaka, 2006)	
OCT4	F: CCCCAGGGCCCCATTTTGGTACC	
SOX2	F: GGCACCCCTGGCATGGCTCTTGGCTC	
KLF4	F: ACGATCGTGGCCCCGGAAAAGGACC	
c-MYC	F: CAACAACCGAAAATGCACCAGCCCCAG	
pMX-AS3200	R: TTATCGTCGACCACTGTGCTGCTG (for OSK)	
pMX-L3205	R: CCCTTTTTCTGGAGACTAAATAAA (for M)	
	**Pluripotency genes (endogenous)**	
OCT4	F: GACAGGGGGAGGGGAGGAGCTAGG<br>R: CTTCCCTCCAACCAGTTGCCCCAAAC	144
SOX2	F: GGGAAATGGGAGGGGTGCAAAAGAGG<br>R: TTGCGTGAGTGTGGATGGGATTGGTG	151
KLF4	F: ATTGGACCCGGTGTACATTC<br>R: AGCACGAACTTGCCCATC	72
c-MYC	F: GCGTCCTGGGAAGGGAGATCCGGAGC<br>R: TTGAGGGGCATCGTCGCGGGAGGCTG	328
NANOG	F: AGTCCCAAAGGCAAACAACCCACTTC<br>R: TGCTGGAGGCTGAGGTATTTCTGTCTC	161
REX1	F: TCTGAGTACATGACAGGCAAGAA<br>R: TCTGATAGGTCAATGCCAGGT	62
GAPDH	F: CAGAACATCATCCCTGCCTCTAG<br>R: TTGAAGTCAGAGGAGACCACCTG	251

Primer sequences of miRNAs analyzed in qRT-PCR

**miRNA**	**Accession**	**Forward primer sequence (5’ – 3’)**
miR-362-5p	MIMAT0000705	GGAATCCTTGGAACCTAGGTGTGAGT
miR-500a-3p	MIMAT0002871	ATGCACCTGGGCAAGGATTCTG
miR-532-3p	MIMAT0004780	CCTCCCACACCCAAGGCTTGCA
miR-125b-5p	MIMAT0000423	GGTCCCTGAGACCCTAACTTGTGA
miR-199a-3p	MIMAT0000232	GCGACAGTAGTCTGCACATTGGTTA
miR-150-3p	MIMAT0004610	CTGGTACAGGCCTGG GGGACA G
RNU6	X07425	F: CTCGCTTCGGCAGCACA<br>R: AACGCTTCACGAATTTGCGT
