# Supplementary material for: Incomplete cellular reprogramming of colorectal cancer cells elicits an epithelial/mesenchymal hybrid phenotype
Source: J Biomed Sci. 2018 Jul 19;25:57. doi: 10.1186/s12929-018-0461-1 (PMC6052640; doi:10.1186/s12929-018-0461-1)
Supplement: Supplementary file 2 — Table S2. Regulated expression of miRNAs known to modulate cellular reprogramming. (PDF 38 kb) [file 12929_2018_461_MOESM2_ESM.pdf]

Additional file 2: Table S2. Regulated expression of miRNAs known to modulate cellular reprogramming

miRNA	Family	Log_2(fold change) iPC vs CRC	Log_2(fold change) iPC vs ESC
Cluster I			
miR-130a-3p	*mir-130*	4.6461377	-5.80656
miR-302a-3p	*mir-302*	-0.8402815	-13.211192
miR-302a-5p		-0.8402815	-11.8833275
miR-302b-3p		-0.8402815	-12.11458
miR-302b-5p		-0.8402815	-6.5738697
miR-302c-3p		-0.8402815	-12.443087
miR-302c-5p		-0.8402815	-7.6779056
miR-302d-3p		-0.8402815	-11.94684
miR-498	*C19MC*	-0.8402815	-5.9553742
miR-512-3p		-0.8402815	-7.684702
miR-512-5p		-0.8402815	-5.0344677
miR-515-3p		-0.8402815	-4.8008533
miR-515-5p		-0.8402815	-6.094796
miR-516b-5p		-0.8402815	-5.8819146
miR-518b		-0.8402815	-5.0204396
miR-518c-5p		-0.8402815	-4.878671
miR-519b-3p		-0.8402815	-6.20168
miR-519c-3p		-0.8402815	-4.9577136
miR-519d-3p		-0.8402815	-5.7168207
miR-519e-5p		-0.8402815	-4.8293204
miR-520a-5p		-0.8402815	-4.816074
miR-520b		-0.8402815	-4.5597577
miR-520c-3p		-0.8402815	-7.3049417
miR-520f-3p		-0.8402815	-5.131268
miR-520g-3p		-0.8402815	-5.0725846
miR-520h		-0.8402815	-3.9666684
miR-525-5p		-0.8402815	-6.0795727
miR-526b-5p		-0.8402815	-4.980953
Cluster II			
miR-642a-3p	*mir-642*	0.71249723	-1.4479289
miR-3162-5p	NA	0.50024843	-1.0586226

Cluster III			
let-7a-5p	*let 7*	0.316	5.9331636
let-7b-5p		0.159	6.4127464
let-7c-5p		0.465	5.9936905
let-7d-5p		-0.442	5.295085
let-7f-5p		0.0166	5.6159344
let-7g-5p		-0.269	6.2615
let-7i-5p		-0.487	2.7759283
miR-181a-5p	*mir-181*	-0.6985439	6.718727
miR-200a-3p	*mir-200*	-0.6929922	4.7573786
miR-200b-3p		-0.8206234	4.6905313
miR-200c-3p		-0.029519081	5.6546283

NA, not available.
