# Supplementary material for: Incomplete cellular reprogramming of colorectal cancer cells elicits an epithelial/mesenchymal hybrid phenotype
Source: J Biomed Sci. 2018 Jul 19;25:57. doi: 10.1186/s12929-018-0461-1 (PMC6052640; doi:10.1186/s12929-018-0461-1)
Supplement: Supplementary file 3 — Table S3. Full list of 102 differentially expressed miRNAs in 4 iPCs vs 2 CRCs. (PDF 220 kb) [file 12929_2018_461_MOESM3_ESM.pdf]

Additional file 3: Table S3. Full list of 102 differentially expressed miRNAs in 4 iPCs vs 2 CRCs

No.	Systematic name[a]	mirBase accession no[b]	Chromosome[c]	miRNA family[d]	FC ([iPC] vs [CRC])[e]	Log_2 FC ([iPC] vs [CRC])[f]	*P* (Corr)[g]
**A. Up-regulated miRNAs (n=52)**							
1.	hsa-miR-30a-5p	MIMAT0000087	6q13	mir-30	15.365	3.942	0.0004
2.	***hsa-miR-125a-3p***	***MIMAT0004602***	***19q13.41***	***mir-10***	***118.546***	***6.889***	***0.0000004***
3.	***hsa-miR-125b-5p***	***MIMAT0000423***	***11q24.1***	***mir-10***	***471.473***	***8.881***	***0.0000004***
4.	hsa-miR-130a-3p	MIMAT0000425	11q12.1	mir-130	25.04	4.646	0.0001
5.	hsa-miR-132-3p	MIMAT0000426	17p13.3	mir-132	18.361	4.199	0.0005
6.	hsa-miR-135a-3p	MIMAT0004595	3p21.1	mir-135	15.596	3.963	0.0006
7.	***hsa-miR-150-3p***	***MIMAT0004610***	***19q13.33***	***mir-150***	***57.917***	***5.856***	***0.000004***
8.	hsa-miR-152-3p	MIMAT0000438	17q21.32	mir-148	18.976	4.246	0.0002
9.	hsa-miR-195-5p	MIMAT0000461	17p13.1	mir-15	15.519	3.956	0.0004
10.	***hsa-miR-199a-3p***	***MIMAT0000232***	***19p13.2***	***mir-199***	***131.654***	***7.041***	***0.0000004***
11.	hsa-miR-199a-5p	MIMAT0000231	19p13.2	mir-199	33.402	5.062	0.00003
12.	hsa-miR-210-3p	MIMAT0000267	11p15.5	mir-210	12.527	3.647	0.01
13.	hsa-miR-371a-5p	MIMAT0004687	19q13.42	mir-290	18.086	4.177	0.0057
14.	hsa-miR-513b-5p	MIMAT0005788	Xq27.3	mir-506	10.414	3.38	0.0032
15.	hsa-miR-652-5p	MIMAT0022709	Xq23	mir-652	17.731	4.148	0.0002
16.	hsa-miR-671-5p	MIMAT0003880	7q36.1	mir-671	36.821	5.202	0.00002
17.	hsa-miR-769-5p	MIMAT0003886	19q13.32	mir-769	32.424	5.019	0.0001
18.	***hsa-miR-1181***	***MIMAT0005826***	***19***	***mir-1181***	***50.918***	***5.67***	***0.00001***
19.	hsa-miR-1185-1-3p	MIMAT0022838	14	mir-154	8.838	3.144	0.0072
20.	hsa-miR-1228-3p	MIMAT0005583	12	mir-1228	26.819	4.745	0.00004
21.	hsa-miR-1249-3p	MIMAT0005901	22q13.31	mir-1249	7.521	2.911	0.0134
22.	hsa-miR-3188	MIMAT0015070	19	mir-3188	23.425	4.55	0.0001
23.	hsa-miR-3911	MIMAT0018185	9	-	6.24	2.641	0.0415
24.	***hsa-miR-3934-5p***	***MIMAT0018349***	***6***	***mir-3934***	***52.355***	***5.71***	***0.0006***
25.	***hsa-miR-4417***	***MIMAT0018929***	***1***	***-***	***74.244***	***6.214***	***0.000002***
26.	hsa-miR-4463	MIMAT0018987	6	-	10.547	3.399	0.0066
27.	hsa-miR-4487	MIMAT0019021	11	-	17.222	4.106	0.0003
28.	hsa-miR-4488	MIMAT0019022	11	mir-4488	30.072	4.91	0.00004
29.	hsa-miR-4532	MIMAT0019071	20	-	11.062	3.468	0.0032
30.	hsa-miR-4646-5p	MIMAT0019707	6	-	26.409	4.723	0.0001
31.	hsa-miR-4651	MIMAT0019715	7	-	31.011	4.955	0.00004
32.	hsa-miR-4655-5p	MIMAT0019721	7	-	27.386	4.775	0.0001
33.	hsa-miR-4690-5p	MIMAT0019779	11	-	15.437	3.948	0.0004
34.	hsa-miR-4695-5p	MIMAT0019788	1	-	21.66	4.437	0.0001
35.	***hsa-miR-4734***	***MIMAT0019859***	***17***	***-***	***82.13***	***6.36***	***0.000002***
36.	hsa-miR-4745-5p	MIMAT0019878	19	-	48.841	5.61	0.00001
37.	hsa-miR-4746-3p	MIMAT0019881	19	-	45.942	5.522	0.00001
38.	hsa-miR-4778-5p	MIMAT0019936	2	-	22.567	4.496	0.0001
39.	hsa-miR-5194	MIMAT0021125	8	-	9.902	3.308	0.0143
40.	hsa-miR-5195-3p	MIMAT0021127	14	-	15.349	3.94	0.0468
41.	hsa-miR-6075	MIMAT0023700	5	-	28.752	4.846	0.00004
42.	hsa-miR-6086	MIMAT0023711	X	-	25.699	4.684	0.0001
43.	***hsa-miR-6723-5p***	***MIMAT0025855***	***1***	***-***	***54.697***	***5.773***	***0.00001***
44.	hsa-miR-6752-5p	MIMAT0027404	11	-	13.193	3.722	0.035
45.	hsa-miR-6757-5p	MIMAT0027414	12	-	35.174	5.136	0.00004

46.	hsa-miR-6778-5p	MIMAT0027456	17	-	26.856	4.747	0.0001
47.	***hsa-miR-6789-5p***	***MIMAT0027478***	***19***	-	***75.413***	***6.237***	***0.000002***
48.	hsa-miR-6798-5p	MIMAT0027496	19	-	15.124	3.919	0.0004
49.	hsa-miR-6808-5p	MIMAT0027516	1	-	26.666	4.737	0.0001
50.	hsa-miR-6867-5p	MIMAT0027634	17	-	26.521	4.729	0.0001
51.	hsa-miR-7152-3p	MIMAT0028215	10	-	29.631	4.889	0.0001
52.	hsa-miR-8089	MIMAT0031016	5q35.3	-	12.588	3.654	0.0017

No.	Systematic name[a]	miRBase accession no[b]	Chromosome[c]	miRNA family[d]	FC ([iPC] vs [CRC])[e]	Log_2 FC ([iPC] vs [CRC])[f]	*P* (Corr)[g]
**B. Down-regulated miRNA (n=50)**							
1.	hsa-miR-7-5p	MIMAT0000252	15q26.1	mir-7	-6.103	-2.61	0.015
2.	hsa-miR-126-3p	MIMAT0000445	9q34.3	mir-126	-11.433	-3.515	0.006
3.	hsa-miR-149-5p	MIMAT0000450	2q37.3	mir-149	-10.262	-3.359	0.006
4.	hsa-miR-181c-5p	MIMAT0000258	19p13.13	mir-181	-9.495	-3.247	0.007
5.	hsa-miR-183-3p	MIMAT0004560	7q32.2	mir-183	-6.82	-2.77	0.014
6.	hsa-miR-192-3p	MIMAT0004543	11q13.1	mir-192	-11.435	-3.515	0.006
7.	***hsa-miR-192-5p***	***MIMAT0000222***	***11q13.1***	***mir-192***	***-71.863***	***-6.167***	***0.005***
8.	hsa-miR-194-3p	MIMAT0004671	11q13.1	mir-194	-8.187	-3.033	0.009
9.	hsa-miR-200a-5p	MIMAT0001620	1p36.33	mir-8	-12.341	-3.625	0.006
10.	hsa-miR-335-3p	MIMAT0004703	7q32.2	mir-335	-6.084	-2.605	0.019
11.	***hsa-miR-338-3p***	***MIMAT0000763***	***17q25.3***	***mir-338***	***-28.578***	***-4.837***	***0.004***
12.	hsa-miR-345-5p	MIMAT0000772	14q32.2	mir-345	-6.706	-2.745	0.014
13.	hsa-miR-362-3p	MIMAT0004683	Xp11.23	mir-362	-12.097	-3.597	0.006
14.	***hsa-miR-362-5p***	***MIMAT0000705***	***Xp11.23***	***mir-362***	***-21.331***	***-4.415***	***0.005***
15.	hsa-miR-421	MIMAT0003339	Xq13.2	mir-95	-9.459	-3.241	0.007
16.	hsa-miR-449b-3p	MIMAT0009203	5q11.2	mir-449	-10.501	-3.392	0.006
17.	hsa-miR-454-3p	MIMAT0003885	17q22	mir-454	-9.29	-3.216	0.007
18.	***hsa-miR-455-3p***	***MIMAT0004784***	***9q32***	***mir-455***	***-24.752***	***-4.629***	***0.004***
19.	hsa-miR-455-5p	MIMAT0003150	9q32	mir-455	-8.804	-3.138	0.008
20.	hsa-miR-500a-3p	MIMAT0002871	Xp11.23	mir-500	-11.588	-3.535	0.006
21.	hsa-miR-500a-5p	MIMAT0004773	Xp11.23	mir-500	-7.206	-2.849	0.012
22.	hsa-miR-505-3p	MIMAT0002876	Xq27.1	mir-505	-10.101	-3.336	0.006
23.	hsa-miR-532-3p	MIMAT0004780	Xp11.23	mir-188	-12.441	-3.637	0.006
24.	***hsa-miR-552-3p***	***MIMAT0003215***	***1p34.3***	***mir-552***	***-17.809***	***-4.154***	***0.005***
25.	hsa-miR-3200-3p	MIMAT0015085	22	mir-3200	-9.81	-3.294	0.007
26.	hsa-miR-3591-3p	MIMAT0019877	18	mir-122	-15.664	-3.969	0.005
27.	hsa-miR-3935	MIMAT0018350	16	-	-12.456	-3.639	0.006
28.	hsa-miR-3940-3p	MIMAT0018356	19	mir-3940	-8.59	-3.103	0.008
29.	hsa-miR-3945	MIMAT0018361	4	-	-11.84	-3.566	0.006
30.	***hsa-miR-4254***	***MIMAT0016884***	***1***	-	***-16.253***	***-4.023***	***0.005***
31.	hsa-miR-4259	MIMAT0016880	1	-	-8.351	-3.062	0.008
32.	hsa-miR-4323	MIMAT0016875	19	-	-8.903	-3.154	0.008
33.	hsa-miR-4652-3p	MIMAT0019717	7	-	-14.167	-3.824	0.005
34.	hsa-miR-4687-5p	MIMAT0019774	11	-	-12.193	-3.608	0.006
35.	***hsa-miR-4725-5p***	***MIMAT0019843***	***17***	-	***-15.874***	***-3.989***	***0.005***
36.	hsa-miR-4769-3p	MIMAT0019923	X	-	-8.859	-3.147	0.008
37.	hsa-miR-6129	MIMAT0024613	17	mir-6129	-14.419	-3.85	0.005

38.	hsa-miR-6730-3p	MIMAT0027362	1	-	-9.713	-3.28	0.007
39.	hsa-miR-6730-5p	MIMAT0027361	1	-	-11.055	-3.467	0.006
40.	hsa-miR-6736-3p	MIMAT0027374	1	-	-8.668	-3.116	0.008
41.	hsa-miR-6737-3p	MIMAT0027376	1	-	-14.51	-3.859	0.005
42.	***hsa-miR-6741-3p***	***MIMAT0027384***	***1***	***-***	***-20.624***	***-4.366***	***0.005***
43.	***hsa-miR-6743-3p***	***MIMAT0027388***	***11***	***-***	***-20.191***	***-4.336***	***0.005***
44.	hsa-miR-6752-3p	MIMAT0027405	11	-	-13.38	-3.742	0.005
45.	hsa-miR-6766-3p	MIMAT0027433	15	-	-13.332	-3.737	0.005
46.	hsa-miR-6779-3p	MIMAT0027459	17	-	-6.66	-2.736	0.014
47.	***hsa-miR-6782-5p***	***MIMAT0027464***	***17***	***-***	***-16.587***	***-4.052***	***0.005***
48.	hsa-miR-6785-3p	MIMAT0027471	17	-	-11.629	-3.54	0.006
49.	hsa-miR-6797-3p	MIMAT0027495	19	-	-10.433	-3.383	0.006
50.	hsa-miR-6803-3p	MIMAT0027507	19	-	-11.134	-3.477	0.006

miRNAs are arranged according to numerical order. MiRNAs in bold and in italics are the top 10 up- or down-regulated miRNAs as shown in **Table 1**. MiRNAs boxed in green are predicted to target the EMT/MET genes as shown in **Table 2**. [a]Systematic name, [b]miRBase accession number and [d]miRNA family are taken from miRBase database; [c]Chromosome locations are taken from HUGO Gene Nomenclature Committee (HGNC); [e]FC: Fold change; [f]Log_2(FC): Log_2(Fold change); [g]P (Corr), corrected *p* value, cutoff at 0.05.
